# Green Synthesis of Aluminum Oxide Nanoparticles Using Clerodendrum phlomidis and Their Antibacterial, Anti-inflammatory, and Antioxidant Activities

**DOI:** 10.7759/cureus.52279

**Published:** 2024-01-14

**Authors:** Srigopika Thanaraj, A.N.K. Mitthun, P. Geetha Sravanthy, Durai Singh Carmelin, Muthuvel Surya, Muthupandian Saravanan

**Affiliations:** 1 Department of Pharmacology, Saveetha Dental College and Hospitals, Saveetha Institute of Medical and Technical Sciences, Chennai, IND

**Keywords:** aluminium oxide, antibacterial, antioxidant, anti-inflammatory, green synthesis

## Abstract

Introduction: *Clerodendrum phlomidis *plays a significant role in many indigenous medical systems, and it can be mostly found in Southeast Asia. The objective of the study was to synthesize and characterize the biosynthesized aluminum oxide nanoparticles (AlO-NPs) using *C. phlomidis *and analyze their antibacterial (bactericidal), antioxidant, and anti-inflammatory activities.

Methods: The extract was prepared by the autoclave-assisted method, and the AlO-NPs were synthesized by the green synthesis method. The biosynthesized AlO-NPs were characterized by ultraviolet-visible (UV-Vis) spectroscopy, Fourier transform infrared (FT-IR), field emission scanning electron microscopy (FE-SEM), and energy dispersive X-ray (EDX) analysis. The antibacterial property was assessed by the Kirby-Bauer well diffusion method, and the antioxidant activity was checked by DPPH (2,2-diphenyl-1-picrylhydrazyl) activity compared with the control L-ascorbic acid. Anti-inflammatory activity was evaluated by an albumin denaturation assay, and diclofenac was used as a control. IBM SPSS Statistics for Windows, Version 21.0 was used for the statistical analysis.

Results: An absorption peak at a wavelength of 380 nm was detected by UV-Vis spectroscopy analysis. It proves that AlO-NPs have been successfully produced by the green synthesis method. The results of the FT-IR study demonstrated the existence of numerous chemicals and functional groups in the 500-3500 cm^-1 ^range. AlO-NPs from the plant extract were subjected to FE-SEM analysis, which revealed an aggregated or spherically cluster-like structure. The sample's elemental makeup, which revealed that it included 38% aluminum and 28% oxygen, was identified with the help of the EDX, and this verified the high purity of the AlO-NPs. The results of the antibacterial activity of AlO-NPs revealed that there was a zone of inhibition for *Enterococcus faecalis; *however, there was no zone of inhibition for *Streptococcus mutans*. The synthesized AlO-NPs exhibit strong antioxidative (DPPH activity) and anti-inflammatory (albumin denaturation assay) action. In this work, the in vitro antioxidant activity of *C. phlomidis *was assessed using the standard, L-ascorbic acid, as a measure of DPPH activity. At a maximum concentration of 500 µg/ml, the obtained results showed the incredible antioxidant properties of the investigated AlO-NPs synthesized from the plant extracts and demonstrated 90% inhibition. AlO-NPs that were biosynthesized showed effective anti-inflammatory activity at a higher concentration of 100 µg/ml and demonstrated 89% inhibition in contrast to the drug diclofenac sodium.

Conclusion: According to the study's findings, AlO-NPs made using a greener synthesis approach have the potential to be used in a variety of industries and are also an affordable and sustainable way to effectively act as anti-inflammatory and antioxidant agents.

## Introduction

*Clerodendrum phlomidis *belongs to theLamiaceae family and is a shrub commonly found in Southeast Asia. Its 7-15 cm long, irregularly branched roots are cylindrical, firm, and yellowish-brown on the surface. Its light yellow wood is difficult to break and has a nearly astringent taste. Its thin bark peels off easily [1]. The roots of *C. phlomidis *are a key component of traditional Ayurvedic formulations like Dashamoolarishta, which has been used since ancient times in the form of kwath or arishta for reducing inflammatory illnesses, pain, and swelling associated with arthritis. For medicinal purposes, Ayurveda suggests using the root bark of *C. phlomidis *rather than the complete root [2]. The plant showed non-toxicity, immunosuppressive function, histamine and arachidonic acid release inhibition, angiotensin-converting enzyme (ACE) inhibition, and HIV-I integrase inhibitory effect [3]. The phytochemical analyses found in *C. phlomidis *were pectolinarigenin, sterol, glycosides, flavones, chalcone, and neo-clerodane. Due to their higher surface area than their bulk counterparts, metal oxide nanoparticles (NPs) are now mostly utilized as heterogeneous nanocatalysts in a range of organic reactions. Aluminum oxide is a chemical compound with the formula Al_2_O_3_, composed of aluminum and oxygen. It is one of the numerous aluminum oxides that occur most frequently, and although it is formally recognized as aluminum (III) oxide, it is more usually referred to as alumina. Many different materials make use of aluminum oxides. Due to its hardness, high melting point, non-volatility, and corrosion resistance, it is widely utilized in ceramics, refractories, and abrasives [4].

Antibiotic resistance has emerged as a significant worldwide concern, while infectious diseases remain the primary cause of death. Pathogens can potentially be fought against through the utilization of novel phytocompounds derived from studies on their hemolytic, antimicrobial, and antioxidant properties. The effectiveness of plant-based phytomedicines in treating infections has increased [5]. The evidence from recent studies shows that plant-based NPs have a high degree of susceptibility to antimicrobial-resistant bacteria. The majority of diseases are also associated with oxidative stress produced by free radicals and metabolic biological processes. Phytochemical compounds possessing various properties were researched for different biomedical applications [6]. The green synthesis method proposed an alternative approach to reduce the usage of hazardous chemical compounds for synthesizing NPs in a sustainable, affordable, and environmentally friendly way. Additionally, phytochemical compounds present in the plant extract can also serve as capping and reducing agents for NP synthesis. The effectiveness of the NPs against *Streptococcus mutans *and *Enterococcus faecalis *and the green synthesis of aluminum oxide nanoparticles (AlO-NPs) utilizing the extract of *C. phlomidis *were examined. The ultraviolet-visible (UV-Vis), Fourier transform infrared (FT-IR), field emission scanning electron microscopy (FE-SEM), and energy dispersive X-ray (EDX) were used to characterize the produced NPs.

## Materials and methods

Sample collection and preparation

**Figure 1:**
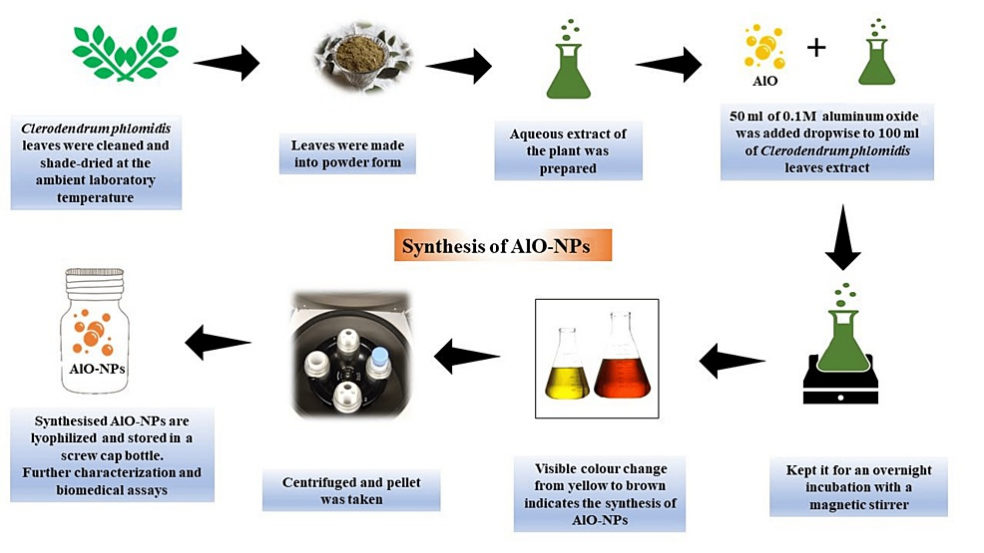
Sample preparation and synthesis of AlO-NPs. AlO-NPs: aluminum oxide nanoparticles.

The leaves of *C. phlomidis* were collected on the campus of Saveetha Institute of Medical and Technical Sciences (SIMATS), Tiruvallur district, Tamil Nadu, India, and their authenticity was confirmed by the botanist Dr. N. Shiva, Assistant Professor, Department of Botany, Raja Doraisingam Government Arts College, Sivagangai, Tamil Nadu. He also verified the sample's taxonomic identification (*C. phlomidis *Linn. (MDCBOT 115/2023)). The leaves of *C. phlomidis *were cleaned, then shade dried at the ambient laboratory condition (25-28°C), and crushed into a powder using a mechanical grinder (Nanchang Kay Xin Yue Technologies Co., Jiangxi, China). Twenty grams of leaf powder were mixed with 100 ml of distilled water and then an autoclave-assisted method was used to prepare the aqueous extract of the plant. Using the titration method, 50 ml of 0.1 M AlO was added dropwise to 100 ml of *C. phlomidis* leaf extract and kept overnight with a magnetic stirrer. There is a visible color change that indicates the synthesis of AlO-NPs, which are then lyophilized and stored in a screw cap bottle for further characterization and biomedical application (Figure 1) [7].

Characterization methods

UV spectroscopy (Labman Double Beam UV-Vis spectrophotometer LMSPUV1900S, India, 190-1100 nm) was used to characterize the production of AlO-NPs by examining them at T0 and T24 between 190 and 1100 nm. FTIR (Bruker Alpha II, Germany) was used to examine the bonding between the functional group and the metal ions at room temperature using the range 500-3500 cm^-1^. The aim was to determine the potential biomolecules responsible for the capping of AlO-NPs and reducing the metal precursors. FE-SEM (JEOL-800S) was used to observe the morphological characteristics of the compounds. FE-SEM and EDX (OXFORD X-Plor-30/C-Swift) analysis were both used to understand the elemental composition of the NPs.

Antibacterial activity

The synthesized AlO-NPs were significantly screened for their antibacterial potential against two bacterial isolates *S. mutans* and *E. faecalis*. The antibacterial potential was assessed by determining the inhibition zone diameter. The sensitivity of bacterial strains toward antibiotics with a clear zone around the well is tested using the well diffusion method [8]. A sterile swab wet with the bacterial suspension was used to apply the inoculum containing the bacterial culture that would be tested to nutrient agar plates. About 6 mm diameter wells were made in an agar medium and added with different concentrations of AlO-NPs (20, 30, 40, 50, 60, and 80 µg/ml), and streptomycin (30 µg/ml) was used as a positive control. The plates were then incubated for 24 hours at 37℃ while standing in an upright position.

Anti-inflammatory activity

The albumin denaturation method with slight changes was used to test anti-inflammatory activity. The reaction mixture included (80, 60, 40, and 20 µg/ml) of bovine serum albumin and (20, 40, 60, 80, and 100 µg/ml) of AlO-NPs in various concentrations. As a negative control drug, a similar volume of dimethyl sulfoxide (DMSO) was utilized. The reaction mixture was incubated for 15 minutes at 37°C, followed by an additional 20 min at 55°C. The absorbance was then measured at 660 nm. At the same absorbance, standard diclofenac sodium was used at final concentrations of (20, 40, 60, 80, and 100 µg/ml) [9]. To calculate inhibition, we used the formula below. The standard deviation was computed using IBM SPSS Statistics for Windows, Version 21.0 (Released 2012; IBM Corp; Armonk, New York, United States) after the mean of the triplicate results.

% of inhibition = (control OD of sample - control OD of control)/control OD of control * 100

In vitro antioxidant activity

This approach was used to investigate DPPH radical scavenging activity. Different concentrations of AlO-NP and ascorbic acid were generated. 2.96 ml of the 0.1 mM DPPH solution was added to 3 ml of the obtained solution. The blend was properly agitated before being placed in the dark room for 20 minutes of incubation. The Shimadzu UV-2450 spectrophotometer, Japan, was used to measure the absorbance of the reaction mixture at 517 nm. Ascorbic acid was utilized as a standard solution, and 0.1 mM DPPH was used as a control [10]. The standard deviation was computed using IBM SPSS Statistics for Windows, Version 21.0 (Released 2012; IBM Corp; Armonk, New York, United States) after the mean of the triplicate results.

## Results

UV-Vis spectroscopy

**Figure 2:**
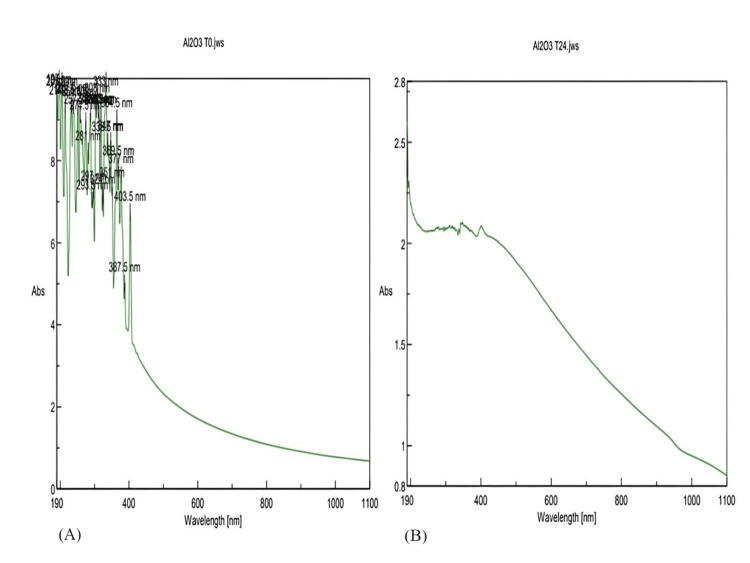
UV-Vis spectroscopy analysis of AlO-NPs at (A) zero time (T0) intervals and (B) after 24 hours (T24). UV-Vis: ultraviolet-visible; AlO-NPs: aluminum oxide nanoparticles.

The synthesized AlO-NPs were characterized for their basic studies and were observed to have properties. The UV-Vis spectrophotometer of the AlO-NPs was analyzed, and absorbance was read. Figure 2 shows the results of the UV-Vis spectrophotometer at zero time (T0) and after a 24-hour (T24) interval. The peak was formed at 380 nm in T24, confirming the formation of AlO-NPs.

Fourier transform infrared spectroscopy

**Figure 3:**
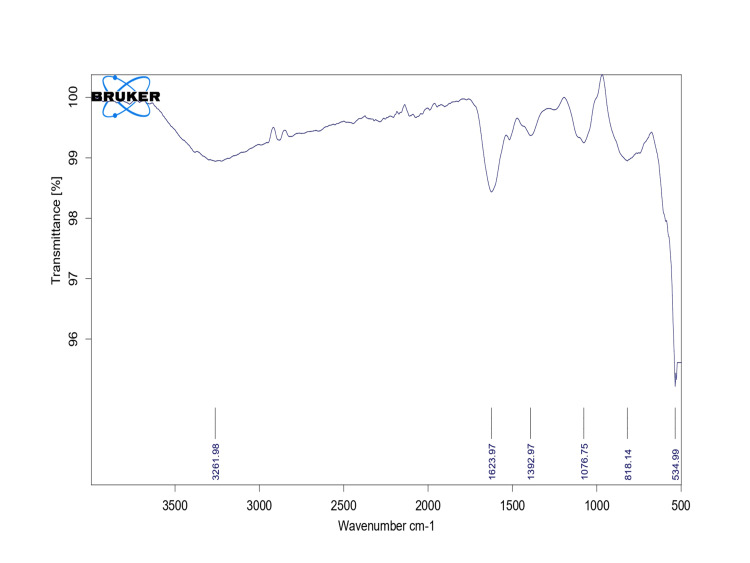
FT-IR spectral analysis of synthesized AlO-NPs. FT-IR: Fourier transform infrared; AlO-NPs: aluminum oxide nanoparticles.

The mixture of synthesized AlO-NPs was added via FT-IR to generate an infrared absorption spectrum, which allowed researchers to determine the chemical bonds in molecules. FT-IR spectrum analysis of biosynthesized AlO-NPs revealed a spectral range between 500 and 3500 cm^-1^ with the absorption peaks shown in the FT-IR (Figure 3). The presence of H-H-bonded O-H stretching and the presence of the Hydroxy group resulted in an absorption peak at 3261.08 cm^-1^. The peak at 1623.97 cm^-1^ is responsible for C=C stretching and the presence of alkenyl. The peak at 1382.97 cm^-1^ is responsible for the presence of the chemical compound aliphatic nitro compounds. Other peaks arise at 1076.35 cm^-1^ (C-O stretch; compound cyclic ethers), 815.14 cm^-1^ (nitrate ion), and 534.99 cm^-1 ^(C-I stretch; aliphatic iodo compounds).

FE-SEM analysis

**Figure 4:**
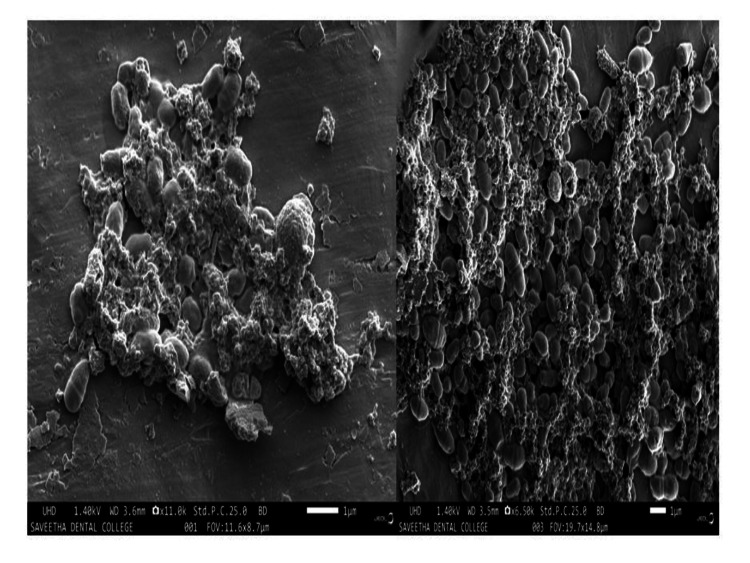
SEM image of synthesized AlO-NPs. SEM: scanning electron microscopy; AlO-NPs: aluminum oxide nanoparticles.

SEM provides an abundance of knowledge regarding the microstructure of nanomaterials, including thin films and nanopowders. Additionally, the signals from the sample can be used to find out more about the materials and structure's composition. SEM analysis of the AlO-NPs from the plant extract showed that they are accumulated or spherically cluster-like in structure with a size range between 80 and 90 nm (Figure 4).

EDX analysis

**Figure 5:**
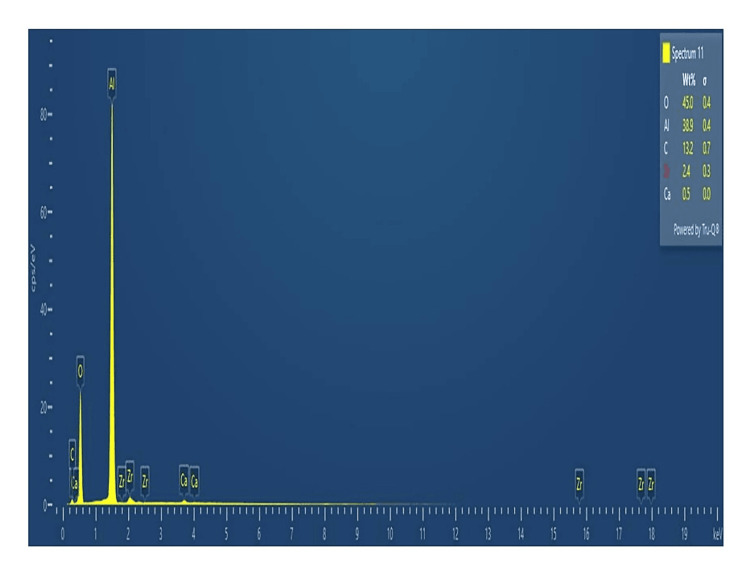
EDX spectral analysis of synthesized AlO-NPs. EDX: energy dispersive X-ray; AlO-NPs: aluminum oxide nanoparticles.

The elemental makeup of the EDX was examined simultaneously with the SEM. A portion of the energy received by a sample is released as core-shell electrons when the sample is activated by an energy source. The energy difference is then emitted as an X-ray with a particular wavelength determined by the atoms from which it started. This happens when an outer shell electron with greater energy fills the space. As a result, the composition of a sample volume activated by an energy source can be determined. Figure 5 depicts the high peaks observed at 82% for aluminum and 28% for oxygen. Then, it confirms that the sample is AlO-NPs.

Antibacterial activity

**Figure 6:**
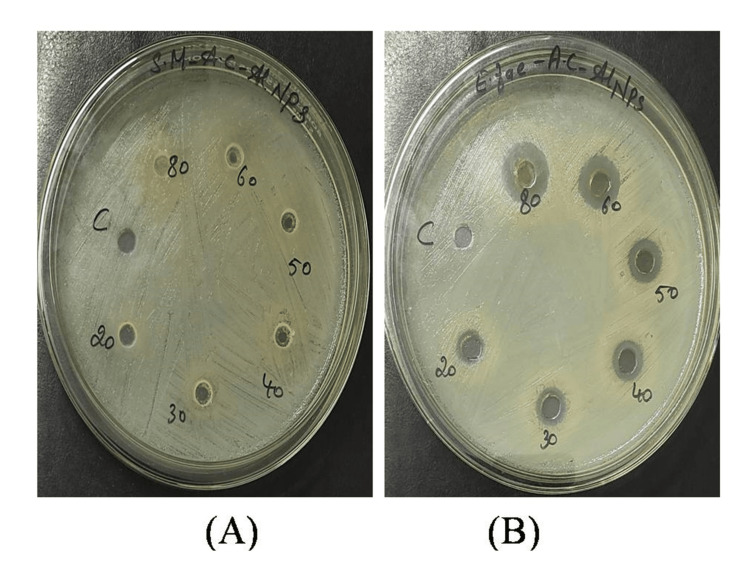
Antibacterial efficacy of synthesized AlO-NPs at different concentrations against (A) S. mutans and (B) E. faecalis. AlO-NPs: aluminum oxide nanoparticles.

The biosynthesized AlO-NPs antibacterial efficacy against *S. mutans *and *E. faecalis *was examined. Different concentrations of biosynthesized NPs made from plant extract (20, 30, 40, 50, 60, 80 µg/ml) were used to observe the zone of inhibition against organisms. The antibacterial activity of the biosynthesized AlO-NPs from plant extract showed a maximum zone of inhibition for *E. faecalis *than that of the positive control and *S. mutans *did not exhibit any zone of inhibition when exposed to synthesized AlO-NPs (Figure 6).

In vitro antioxidant activity (DPPH activity)

**Figure 7:**
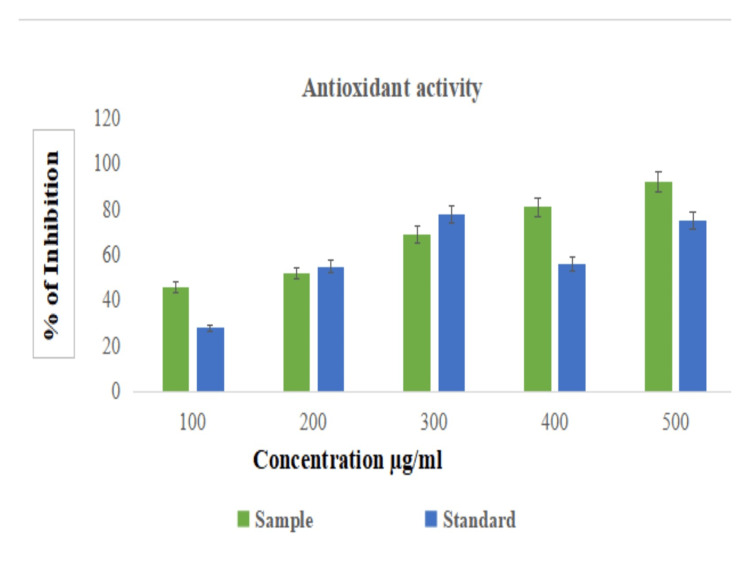
Antioxidant activity of AlO-NPs compared with standard (L-ascorbic acid). AlO-NPs: aluminum oxide nanoparticles.

The biosynthesized AlO-NPs demonstrated in vitro DPPH radical scavenging activities in a dose-dependent manner (100, 200, 300, 400, and 500 µg/ml), and they showed significant antioxidant activity at 500 µg/ml compared with the standard (L-ascorbic acid) (Figure 7).

In vitro anti-inflammatory activity (albumin denaturation assay)

**Figure 8:**
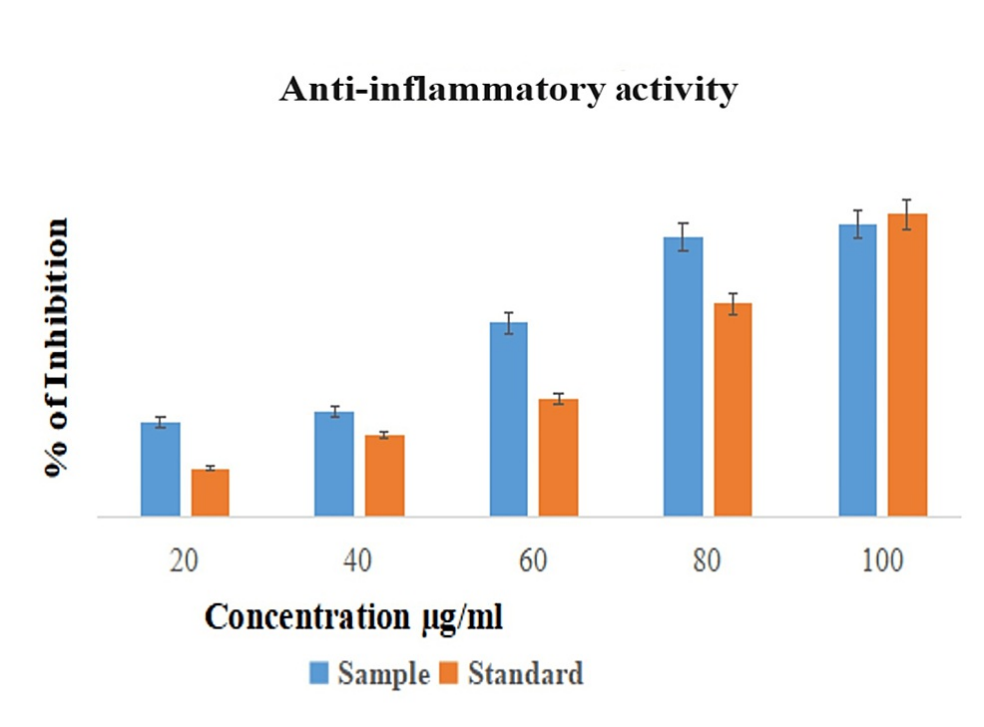
Anti-inflammatory activity of AlO-NPs compared with the standard (diclofenac). AlO-NPs: aluminum oxide nanoparticles.

The result was observed at various concentrations (20, 40, 60, 80, and 100 µg/ml) of AlO-NPs, and it showed significant anti-inflammatory activity (95%) at 100 µg/ml concentration compared to standard diclofenac drug (90%) (Figure 8). There are many phytochemical compounds present in the plant aqueous extract that are mainly responsible compounds for the anti-inflammatory activity.

## Discussion

*Clerodendrum phlomidis* extract, a naturally occurring harmless hydrocolloid and green technology, was used to biosynthesize AlO-NPs. The biosynthesized AlO-NPs were visually confirmed by the color change of the nanoparticles, which changed from light yellow to brown precipitate. The phytochemical compounds present in *C. phlomidis *are responsible for the reduction and act as a stabilizing agent. The indication of color change confirms the presence of AlO-NPs. The AlO-NPs were characterized by UV-Vis spectroscopy, and the peak was formed at 380 nm in T24. Compared to a similar study by Saleh et al. [11], the major peak was observed at 264 nm, confirming the formation of AlO-NPs. According to the literature, the posture of absorption varies depending on the samples utilized for generating AlO-NPs, such as the macroalgae *Sargassum ilicifolium* extract (227 nm) and *Citrus aurantium* L. extract (322 nm) [12], and Al foil waste (237 nm) [13].

According to Khan et al. [14], an increase or decrease in absorbance influences the size of the particle. Their optical absorbance increases as a result of AlO-NPs. AlO NPs' optical energy gap was similarly found to be decreasing as particle size and agglomeration increased [15]. The existence of functional groups has been shown by the FT-IR data. When compared to the study of AlO-NPs synthesized from aluminum foil, the characterization of FT-IR results showed that O-H stretching is specifically present at 3457 cm^-1 ^and 3438 cm^-1^ [16]. In this case study, O-H stretching was present at 3261.08 cm^-1^. The synthesized NPs contained multiple peaks in that range, probably a result of contaminants and reagents utilized during preparation. SEM analysis showed the spherical-shaped structure, and this structural analysis of nanoparticles is compared with the previous study of Al-NP using *Aerva lanata *extract, which revealed the appearance of the spherical-shaped structure indicated by SEM analysis. It is clear that spherical AlO-NPs between the sizes of 50 nm and 70 nm exist [17], and it is also obvious that producing ultrafine, monodispersed alumina NPs at calcination temperatures between 800°C and 1000°C is crucial for many applications. Thus, a similar structure of SEM analysis morphologically confirms the AlO-NPs.

The antibacterial activity of the biosynthesized AlO-NPs from plant extract showed a maximum zone of inhibition for *E. faecalis*. It was shown that *E. faecalis* was extremely vulnerable to AlO-NPs. Metal cation release disrupts the functionality of proteins and cell structures [18]. The electrostatic interaction of AlO-NPs with the bacterial outer membrane or cell wall and the creation of nano Al^3+^ ions are the primary mechanisms of their antimicrobial effect. Nicotinamide adenine dinucleotide phosphate (NADPH) then activates these nano Al^3+^ ions to form reactive oxygen species (ROS), which in turn causes oxidative DNA damage, lipid membrane oxidation, and protein denaturation. Moreover, it results in cell death [19]. *Streptococcus mutans *did not exhibit any zone of inhibition when exposed to synthesized AlO-NPs because *S. mutans* can create biofilms. It was challenging for the AlO-NPs to adhere to the bacterial cell wall surface. The zone of inhibition of the different extracts was observed and measured. The zone of inhibition of the bacterial growth and the zone with a diameter of >6 mm were considered to be as susceptible and a zone with a diameter <6 mm was considered to be as resistant.

The plant-based nanomaterials have antibacterial activity, similar to that of AlO-NPs, but with less toxicity. The results showed that higher concentrations of AlO-NPs led to increased antibacterial activity, consistent with earlier study findings [20].

A dose-dependent pattern of antioxidant activity is observed. The antioxidant activity increases along with the dosage concentration. When compared to the standard drug, AlO-NPs had a higher concentration. The results of this current study showed that DPPH scavenging activity is indicated by a distinctive color change from blue to yellow at 517 nm and exhibited a concentrated-dependent increase [21] in absorbance values of the biosynthesized AlO-NPs of *C. phlomidis*. Compared to a similar study, DPPH has an absorption band at 515 nm, which disappears upon reduction by an antiradical compound [22]. The absorption band of DPPH is located at 515 nm and can be scavenged by the variety of phytochemical compounds present in the plant extract such as sterol, glycosides, flavones, chalcone, and neo-clerodane.

Anti-inflammatory activity showed maximum activity at 100 µg/ml and minimum activity at 20 µg/ml. When compared to the standard drug (diclofenac) [23], the metal NPs enhance the anti-inflammatory process and the way NPs interact with cells. It is a beneficial response to an injury or wound caused by an infection. According to the current results, *C. phlomidis* contains many phytochemical compounds (flavanones, pectolinarigenin, sterol, glycosides, flavones, chalcone, and neo-clerodane), and it can be a potent therapeutic agent for the treatment of acute inflammations.

Limitations

In the current study, we reported several types of in vitro analyses to evaluate the AlO-NPs synthesized from the plant extract. Additional in vivo research, such as animal and clinical trials, will be helpful in better understanding its effects.

## Conclusions

AlO-NPs from the aqueous extract of *C. phlomidis *were synthesized using the green synthesis method and confirmed visually by the formation of a brown precipitate due to color change. Further, it was confirmed by characterization methods such as UV-Vis, FT-IR, FE-SEM, and EDX. Our synthesized AlO-NPs were tested for antibacterial efficacy against *S. mutans* and *E. faecalis, *showing significant antibacterial efficacy against* S. mutans*. Finally, the findings demonstrated that biosynthesized AlO-NPs have strong anti-inflammatory and antioxidant properties. Further research is needed to fully understand the biological potential of AlO-NPs, both in vitro and in vivo. Furthermore, laboratory testing and clinical trials are suggested to analyze the therapeutic efficacy of synthesized AlO-NPs used as a drug. Therefore, the aforementioned AlO-NPs are being considered for biomedical applications, such as pharmaceuticals and medicines.
